# Microbes

**Published:** 1889-11-02

**Authors:** 


### MICROBES.

With regard to the treatment of diseases depending on microbes in the intestines, Dr. Brunton remarks that the indications are both to remove the microbes and the poisons they have formed, to neutralise the injurious effects of any poisons formed, and to lessen the growth of any microbes which may have remained in the intestines. First, there is the eliminative treatment. The treatment of diarrhœa by purgatives is still largely followed and with great advantage. If we try to lessen diarrhœa by chalk or opium we make matters worse, whereas a dose of castor oil, with a few drops of laudanum in it just sufficient to lessen irritation, will often put an end to the attack by the expulsion of the microbes and the poisons they form. But intestinal disinfectants are also desirable, and a disinfectant is necessary which has no injurious effects on the organism. "The diphenyl compounds seem to have a less toxic action than those containing a single phenyl group, and possibly the best direction to look for new intestinal disinfectants would be among the sulpho-compounds of the diphenyl group." In this connection ortho-phenol-sulphonic acid, or aseptol, is mentioned as an intestinal disinfectant. The commercial aseptol is a solution containing one part in three, and it is administered internally in the same dose as salicylic acfd in cases of intestinal catarrh. But another indication in the treatment is "starving out" the microbes. For this reason in infantile diarrhœa the administration of milk may be entirely stopped with benefit, as it affords nourishment to the bacteria, and is decomposed by them into poisonous substances.

